# Subclinical Hypothyroidism and Depression: A Systematic Review and Meta-Analysis

**DOI:** 10.3389/fendo.2019.00340

**Published:** 2019-06-04

**Authors:** Rong Tang, Jian Wang, Lili Yang, Xiaohong Ding, Yufan Zhong, Jiexue Pan, Haiyan Yang, Liangshan Mu, Xia Chen, Zimiao Chen

**Affiliations:** ^1^Department of Endocrinology, The First Affiliated Hospital of Wenzhou Medical University, Wenzhou, China; ^2^Department of Hand Surgery and Peripheral Neurosurgery, The First Affiliated Hospital of Wenzhou Medical University, Wenzhou, China; ^3^Department of Radiology, The Second Affiliated Hospital and Yuying Children's Hospital of Wenzhou Medical University, Wenzhou, China; ^4^The First Clinical Medical School, Wenzhou Medical University, Wenzhou, China; ^5^The Second Clinical Medical School, Wenzhou Medical University, Wenzhou, China; ^6^Reproductive Medicine Center, The First Affiliated Hospital of Wenzhou Medical University, Wenzhou, China

**Keywords:** subclinical hypothyroidism, depression, thyroid, neuroendocrinology, thyroxine

## Abstract

**Background:** Thyroid function is closely associated with neuropsychological functions, including mental state and cognitive functions. Although thyroid function is routinely examined in persons with depressive symptom, the association between subclinical hypothyroidism (SCH) and depression remains inconclusive.

**Objective:** This systematic review and meta-analysis aimed to evaluate the risk of depression in persons with SCH.

**Methods:** The PubMed, Embase, and Web of Science databases were searched up to August 2018. The primary outcome was the prevalence of depression, as evaluated by various types of self-reported depression scales. Odds ratios (ORs) were calculated to compare the risk of depression between persons with SCH and those with euthyroidism.

**Results:** Twenty-one studies were included in the systematic review, with a total of 103,375 subjects from 7 studies being pooled for the meta-analysis to evaluate the risk of depression. The meta-analysis showed that persons with SCH had a significantly elevated risk of depression than persons with euthyroidism (OR = 1.78, 95% confidence interval [CI]: 1.11–2.86, *P* = 0.02). No publication bias was found, as indicated by Egger's test (*t* = −0.49, *P* = 0.647) and Begg's test (*z* = −0.15, *P* = 0.881). In addition, the funnel plot showed a symmetric distribution.

**Conclusions:** This meta-analysis demonstrated that SCH was positively associated with the risk of depression, especially in persons above 50 years of age, suggesting it is necessary to pay close attention to depressive symptoms in persons with SCH.

## Introduction

Thyroid function is closely associated with neuropsychological functions, including mental state and cognitive functions (1). Subclinical hypothyroidism (SCH) is defined as a condition with elevated thyroid-stimulating hormone (TSH) and normal free thyroxine (T4) levels (2). The prevalence of SCH is approximately 4–10% in adults (3) and is associated with neuropsychological dysfunction (4).

In recent years, an increased number of studies investigated the association between SCH and depression (5, 6). However, the findings of these reports could not reach a consensus. Some studies reported that the prevalence of depression is higher in persons with SCH than in euthyroid people, (4, 7) while others found the risk for depression is comparable between the two population (6, 8). Therefore, this systematic review aimed to summarize the existing information and evaluate the risk of depression in persons with SCH.

## Methods

### Study Design

This meta-analysis was performed according to the Preferred Reporting Items for Systematic Reviews and Meta-Analysis (PRISMA) (9) and the Meta-analysis of Observational Studies in Epidemiology (MOOSE) (10).

### Participants

SCH participants were diagnosed according to the specific diagnostic criteria in each included study (Table 1) and the controls for these studies included participants with euthyroid function. In addition, all participants were not limited by age group, geographical region, or race.

**Table 1:** Characteristics of included studies.

**Study**	**Study design**	SCH mean age^*^ (Years)	**Controls mean age (Years)**	**Country**	**Scales for depression**	**Definition for SCH**	**Conclusions**
Blum et al. (11)	Cohort	75.66 ± 3.4	74.96 ± 3.2	Dutch	GDS	TSH ≥ 4.5 mIU/L, fT4 12–18 pmol/L	NA
Jorde et al. (12)	Cohort	62.5 ± 11.7	61.0 ± 12.5	Norway	GHQ-30	TSH 3.5–10.0 mIU/L, fT3 and T4 were normal	NA
Kim et al. (6)	Cohort	39.8 ± 6.6	30.7 ± 7.2	South Korea	CES-D	TSH ≥ 5 mIU/L, fT4 0.93–1.7 ng/dL	NA
Park et al. (13)	Cross-sectional	76.8 ± 9.0	76.5 ± 9.0	Korea	GDS-K	TSH ≥ 4.1mIU/L, fT4 0.7–1.8 ng/dL	NA
Baldini et al. (14)	Cohort	55.2 ± 8.8	50.3 ± 9.2	Italy	HRSD	TSH ≥ 4.6 mIU/L, fT3 2.6–5.6 pg/mL, fT4 6.3–15.3 pg/mL	NA
Gussekloo et al. (15)	Cohort	85	85	Netherlands	HADS	TSH > 4 mIU/L, fT3 and T4 were normal	NA
Engum et al. (16)	Cohort	40–89	40–89	Norway	HADS	TSH > 4 mIU/L, fT3 and T4 were normal	NA
Fjaellegaard et al. (5)	Cross-sectional	42–63	43–63	Denmark	MDI	TSH ≥ 4.6 mIU/L, fT3 2.6–5.6 pg/mL, fT4 6.3–15.3 pg/mL	NA
Almeida et al. (17)	Cross-sectional	75.3 ± 4.1	75.3 ± 4.1	Australia	GDS-15	TSH ≥ 4 mIU/L, fT4 10–22 pmoL/L	NA
Roberts et al. (18)	Cohort	≥65	≥65	England	HADS	TSH ≥ 5.5 mIU/L, fT4 9–20 pmoL/L	NA
Pop et al. (19)	Cohort	49.2 ± 2.2	49.2 ± 2.2	Netherlands	EDS	TSH ≥ 6 mIU/L, fT4 8–26 pmoL/L	NA
Demartini et al. (4)	Case-control	62.5 ± 13.4	52.0 ± 14.7	Italy	MADRS	TSH ≥ 4.97 mIU/L, fT3 2.64–5.68 pmoL/L, fT4 9.1–19.6 pmoL	A
Larisch et al. (20)	Cohort	47 ± 12	61 ± 14	Germany	GHQ-12	TSH 4.4–13 mIU/L, fT3 and fT4 were normal	A
Almeida et al. (21)	Cross-sectional	49.1 ± 10.3	44.8 ± 9.6	Brazil	HAMD	TSH ≥ 4 mIU/L, fT4 0.8–1.8 ng/dL	A
Chueire et al. (22)	Cohort	≥60	≥60	Brazil	DSMIV	TSH ≥ 4.5 mIU/L, fT4 0.74–2.1 ng/dL	A
Yu et al. (7)	Case-control	46.53 ± 6.61	45.12 ± 5.34	China	HAMD	TSH ≥4 mIU/L, fT3 and fT4 were normal	A
Gulseren et al. (23)	Case-control	40.9 ± 14.2	40.5 ± 7.6	Turkey	HAM-D	TSH ≥ 5 mIU/L, fT3 3.1–6.8 pmoL/L, fT4 12–22 pmoL/L	A
Vishnoi et al. (24)	Case-control	52.5 ± 11.5	52.4 ± 11.5	India	DSMIV	TSH ≥ 4.5 mIU/L, fT4 0.74–2.1 ng/dL	A
Chueire et al. (25)	Case-control	60–89	60–92	Brazil	CES-D	TSH≥ 4.5 mIU/L, fT3 andT4 were normal	A
Manciet et al. (26)	Cohort	≥65	≥65	France	PHQ-9	TSH ≥ 6.68 mIU/L, fT4 0.89 ± 1.76 ng/dL	NA
Hong et al. (8)	Cross-sectional	47.9 ± 1.8	44.4 ± 0.4	Korea	DSM-IV	TSH ≥ 4.5 mIU/L,fT3 60–200 ng/dL, fT4 4.5–11.5 ug/mL	NA

* *The mean age of the enrolled patients*.

*SCH, subclinical hypothyroidism; TSH, thyroid-stimulating hormone; fT3, free triiodothyronine; fT4, free thyroxine; GDS-15, The Geriatric Depression Scale 15; GHQ-30, The General Health Questionnaire; CES-D, The Center for Epidemiologic Studies Depression scale; GDS-K, The geriatric depression scale; HRSD, Hamilton rating scale for depression; HADS, Hospital Anxiety and Depression Scale; MDI, Major (ICD-10) Depression Inventory; EDS, The Edinburgh Depression Scale; MADRS, Montgomery-Asberg Depression Rating Scale; HAM-D, Hamilton Rating Scale for Depression; GHQ-12, Twelve item general health questionnaire; DSM-IV, Diagnostic and Statistical Manual of Mental Disorders-IV; HDRS, The Hamilton Depression Rating Scale; RDC, The Research Diagnostic Criteria; NA, No association; A, Significant association*.

### Search Strategy

The PubMed, Embase, and Web of Science literature databases were searched, without restriction of language, for publications up to August 2018. In order not to miss potential studies, we use “hypothyroidism” as a keyword instead of “subclinical hypothyroidism” to expand retrieval results because some articles may use similar terms like “mild hypothyroidism” or “slight hypothyroidism.” This searched keyword was combined with “depression” and “depressive symptom” in the “title and abstract” column. The complete search items were “[(hypothyroidism) AND (depression OR depressive symptom)]” in PubMed and Web of Science, and hypothyroidism AND (depression OR “depressive symptom”) in Embase.

### Studies Sections and Data Extraction

Selected studies were eligible if they met the following criteria: (1) SCH was confirmed by elevated TSH levels and circulating thyroid hormones within the normal range; and (2) the study was a cross-sectional, case-control, or cohort study. Studies were excluded if: (1) the title and/or abstract were not appropriate for the objective of this review; (2) the use of antidepressant drugs was not allowed in a specific period before each trial; and (3) the article was a review, case report, non-human study, or an abstract or conference proceeding, or if it contained unpublished data. Two reviewers screened potential articles independently and a third reviewer made the final decision when discrepancies were found with a specific study.

Data, including study name, characteristics of the study population (age, race, and sex), main conclusion(s), diagnostic criteria, number of SCH participants with depression and the total number of SCH patients, the number of euthyroid participants with depression, the total number of euthyroid participants were extracted. If published papers only provided the percentage of depressive symptoms, the evaluator calculated the numbers of participants by the calculator in Revman version 5.3 (The Cochrane Collaboration, Copenhagen, Denmark).

### Statistical Analysis

The literature quality evaluation was conducted by means of the Newcastle-Ottawa Quality Assessment Scale (27). Two reviewers conducted this step independently and disagreements were resolved by consensus. The presence of publication bias was assessed by the combination of Egger's test, Begg's test, and visual inspection of the funnel plot.

Odds ratio (OR) and 95% confidence intervals (CIs) were calculated for the outcome of depression, in which the prevalence of depressive symptoms in SCH participants and a normal population was assessed. The reason for the choice of OR was due to the retrospective design of this meta-analysis based on published studies that varied in design, subject populations, primary outcome measures, and research quality (28).

A forest plot was used to present the pooled OR value, and heterogeneity was evaluated using the *I*^2^ statistic. Due to the potential clinical heterogeneity, a random-effect model was chosen to synthesize data instead of a fixed-effect model. Heterogeneity was considered significant if the *I*^2^ value was over 25%. All reported test results were two-tailed and a *P* value < 0.05 was considered statistically significant. All data analyses were performed with Revman 5.3.

## Results

### Study Selection

**Figure 1:**
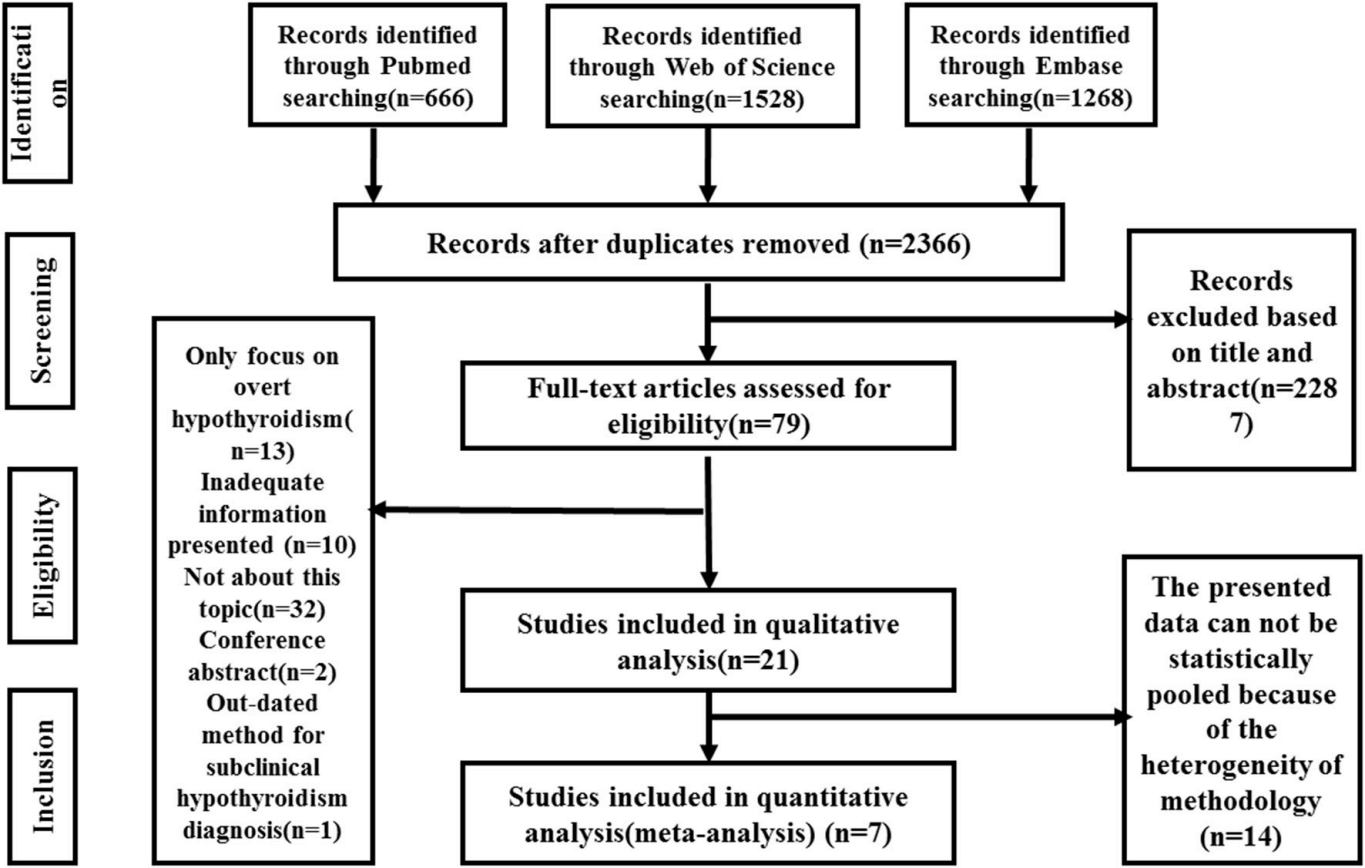
Meta-analysis flow chart. RCT, randomized controlled trial; L-T4, levothyroxine; SCH, subclinical hypothyroidism.

A flow chart of the meta-analysis is shown in Figure 1. Initially, a total of 3,462 studies were identified by the search strategy. After duplicates removal, 2,366 studies received screening based on the title or abstract. Next, 79 studies were assessed for eligibility by reviewing the full-text article. In addition, 58 studies were excluded for the following reasons: only focusing on overt hypothyroidism (*n* = 13); inadequate information presented (*n* = 10); not related to our study topic (*n* = 32); conference abstract (*n* = 2); outdated method for SCH diagnosis (*n* = 1). A total of 21 studies were included in the qualitative analysis. Among these studies, 14 studies presented the mean scores of depression scales of each group or only provided the regression results without details, which hinders access to data analysis. Finally, seven studies were eligible and included in the final quantitative analysis (meta-analysis).

### Characteristics of Included Studies

The characteristics of 21 studies were presented in Table 1, which included 11 cohort, 5 cross-sectional, and 5 case-control studies with mean participant age of 39.8–92 years. Participants in these studies came from different regions, including America, Europe, and Asia. Studies from Korea, the Netherlands, and Norway showed null associations between SCH and depression, while all the studies from Brazil showed significant associations. Heterogeneity among the studies was caused by different SCH diagnosis criteria and variations in the use of depression scales for assessment. Serum TSH cut-off values for the diagnosis of SCH range from 3.5 to 6 mIU/L. Various scales were used for detecting depressive symptoms, including the geriatric depression scale (GDS) (29), the hospital anxiety and depression scale (HADS) (30), and the Hamilton depression rating scale (HAMD) (31). Among these 21 studies, 8 showed significant associations between SCH and depression, while the other 13 did not show this relationship.

### Risk of Bias

The quality of the literature for the analyzed studies was evaluated using the Newcastle-Ottawa Quality Assessment Scale. Overall, the quality for each study was found to be “good” as indicated by 6-plus scores. The main drawback of these studies was that participants in some cohorts were not representative and outcome assessment was self-reported (questionnaires); however, this is typically inevitable for these types of studies. For cross-sectional studies, the common deficiency was the lack of a direct description of response rates and the completeness of data collection that could be determined. The case-control study by Yu et al. (7) also lacked the typical case representation due to the enrolled participants having been diagnosed with Graves' disease before the trial, but the overall score was still intermediate (6 scores).

**Figure 2:**
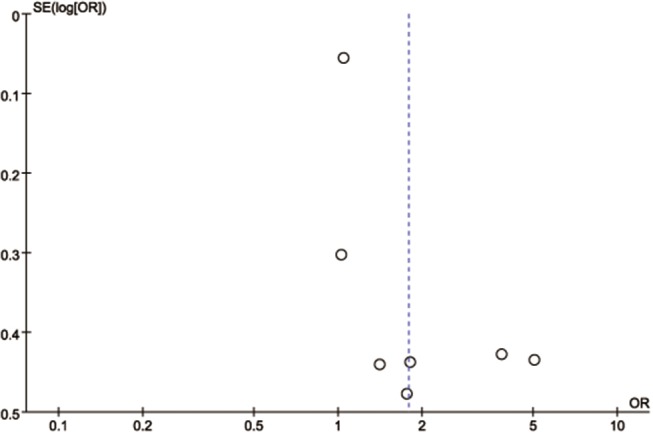
Funnel plot analysis. SE, standard error; OR, odds ratio.

No publication bias was found from Egger's test (*t* = −0.49, *P* = 0.647) or Begg's test (*z* = −0.15, *P* = 0.881), and the funnel plot showed a symmetric distribution (Figure 2). Obvious heterogeneity (*I*^2^ = 75%) was detected and not diminished after a sensitivity analysis by removing a single study at a time in an iterated manner.

### The Risk of Depression in Persons With SCH

**Figure 3:**
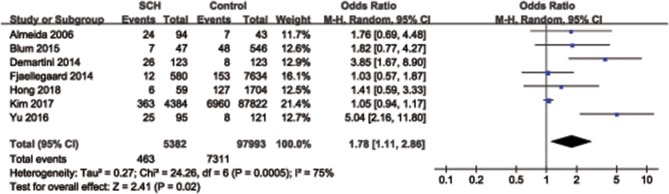
Pooled analysis for subclinical hypothyroidism and depression. SCH, subclinical hypothyroidism; CI, confidence interval.

**Figure 4:**
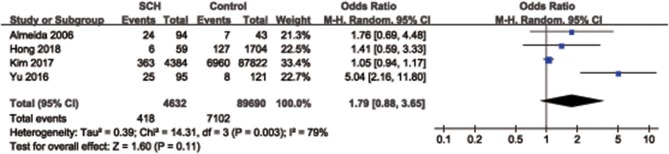
Subgroup analysis for the association between subclinical hypothyroidism and depression in the participants with a mean age of <50 years. SCH, subclinical hypothyroidism; CI, confidence interval.

Overall, a significant difference was found for the synthesized endpoint (OR = 1.78, 95% CI: 1.11–2.86, *P* = 0.02), and the prevalence of depression in SCH participants (8.6%, 463/5,382) was slightly higher than that of the control group (7.5%, 7,311/97,993) (Figure 3). Considering the influence of age on the results, a subgroup analysis including 4 studies, whose participants were characterized with a mean age of < 50 years, was performed. No significant difference was observed for the risk of depression between SCH participants and the control group in participants with a mean age < 50 years (OR = 1.79, 95% CI: 0.88–3.65, *P* = 0.11) (Figure 4).

## Discussion

### Summary of Main Findings

This systematic review and meta-analysis demonstrated that the prevalence of depression is slightly higher in persons with SCH, especially in persons above 50 years of age, suggesting subclinical hypothyroidism might be a risk factor for depression.

Previously, many studies investigated the relationship between SCH and depression; however, these studies showed conflicting conclusions. Recently, a large cohort study conducted by Kim et al. analyzed a total of 92,206 young to middle-aged adults and found no link between SCH and depression (6). In the above study, TSH, free T4, and free T3 were measured, and the Epidemiologic Studies-Depression Scale (CES-D) was used for assessments at least twice a year. However, the limitation of aforementioned study was that the participants primarily were middle-aged and generally healthy men and women from Korea, which may not be generalizable to other ethnic or age groups. An interesting finding in our review was that all studies from Korea (6, 13, 32), the Netherlands (15, 19), and Norway (12, 16) showed no association between SCH and depression, while studies from Brazil (22, 25, 33) did show significant associations, which suggests that regional differences, including economy, lifestyle, and diet, play key roles in these observational studies.

Another interesting phenomenon was observed in terms of age stratification. In our qualitative analysis, we found that 22% (2/9) of the included studies, which enrolled participants aged over 60 years, showed a positive correlation between depression and SCH (4, 22, 25); while 50% (4/8) of the studies, which had participants aged <60 years, reported that the presence of this association (7, 23, 24, 33). However, these qualitative observations are limited by different sample sizes. In order to further investigate whether age influences the risk of depression in SCH participants quantitatively, a subgroup analysis including 4 studies in which the mean participant age was <50 years was conducted, showing no association between depression and SCH. This result indicates that only older participants are more susceptible to SCH. Thus, in elderly populations, depression is more likely to occur in participants with SCH. This phenomenon may depend on the higher reactivity of 5-HT system to the decreased T4 level in elder people compared with young individuals (34).

Fourteen studies were excluded in our final meta-analysis because they utilized mean depressive scores or other methodologies to present their results in the final publication, making it impossible to meta-analyze these data. Among these studies, Park et al. (13) enrolled 944 Korean people aged at least 65 years old, who did not have any thyroid diseases in their study, with neuropsychological function being evaluated by GDS-K score and serum TSH and free thyroxine also being measured. In their study, mean scores of depressive symptoms were comparable between participants with SCH and euthyroid participants. However, a recent study (24) reported the opposite conclusion. In this study, HAM-D scores were significantly higher for participants with SCH as compared to controls. In addition, after 2 months of levothyroxine (L-T4) treatment, they observed a significant fall in TSH levels and HAM-D scores. Several studies showed similar results, illustrating that a “worse” score is more likely in persons with SCH. Therefore, the use of various scales for the detection and quantification of depression may be limited by methodological heterogeneity, disturbing the horizontal comparisons among different studies. We here recommend the CES-D scale for future studies involved in this area (35), which is generally recommended for the primary screening of depression in community care or primary care, as it has shown great sensitivity and specificity in both young and elderly populations (36).

Although some studies reported that L-T4 treatment has the benefit of improving depressive symptoms, the pathophysiological role of thyroid hormones in depression remains unclear (37–40). A biologically plausible theory is that L-T4 may exert modulatory effects on the illness via an increase in serotonergic neurotransmission, specifically by reducing the sensitivity of 5-HT1A (5-hydroxytryptamine 1A) autoreceptors in the raphe area and by increasing 5-HT2 receptor sensitivity (41). High serum TSH levels are associated with depression in the elderly, which suggests that increased TSH could disturb the stability of 5-HT system and thereby facilitates the development of depression in SCH participants (22). This effect is partially explained by the efficacy of L-T4 treatment observed in some studies.

This meta-analysis demonstrates that there is a higher prevalence of depression in persons with SCH, raising some suggestions for clinical practice. First, timely screening and intervention of depressive symptoms are of great importance in persons with SCH. Second, our findings strongly support the routine examination of thyroid function in participants with depression in the clinic. Third, L-T4, which is the suggested treatment for hypothyroidism, may be effective in improving depressive symptoms in participants with SCH if the association between the presence of SCH and depression will be proved. Therefore, well-conducted and large-scale random controlled trials are expected to verify the therapeutic effect on depressive symptoms by L-T4 (12, 37–40, 42–44).

### Strengths and Limitation

To the best of our knowledge, this is the first systematic review and meta-analysis aimed to verify the association between subclinical hypothyroidism and depression. The total number of the cases and controls was sufficiently large to support our conclusions. However, several limitations should be taken into consideration. First, the age of subjects in the included studies was not restricted to a specific range. Second, the included reports were designed as observational studies (case control, cross-sectional, and cohort studies) with different data collection methods (retrospective and prospective). Third, there may be possible bias due to the heterogeneity of the analyzed studies in terms of the definition of SCH and the evaluation of depression. Nonetheless, it is recommended that each laboratory should establish their own cut-off value to evaluate SCH based on the age, region, ethnic factors. Thus, the heterogeneity pertaining to the different cut-off value of TSH for the diagnosis of SCH is acceptable. In addition, SCH may result from antithyroid treatment in patients with Graves' disease. In such patients, neurological disturbances may stem from either the hyperthyroid state or the iatrogenic hypothyroidism that ensues from antithyroid treatment (45). Future studies should focus on the underlying mechanism behind the association between SCH and depression and whether depression or other neuropsychological conditions could be treated through the supplement of L-T4.

### Conclusion

In conclusion, our study demonstrated that depression is positively associated with SCH, suggesting that it is necessary to for clinicians to pay special attention to depression symptoms in patients with SCH. Future studies should focus on whether depression or other neuropsychological conditions could be improved by treating thyroid dysfunction and any potential mechanism.

## Author Contributions

RT, JW, and LY: data analysis. XD and YZ: data extraction. JP, HY, and LM article screening. XC and ZC: paper writing and review.

### Conflict of Interest Statement

The authors declare that the research was conducted in the absence of any commercial or financial relationships that could be construed as a potential conflict of interest.
